# Implementation of advanced Optimum Contribution Selection in small-scale breeding schemes: prospects and challenges in Vorderwald cattle

**DOI:** 10.1017/S1751731119002295

**Published:** 2019-10-10

**Authors:** S. Kohl, R. Wellmann, P. Herold

**Affiliations:** 1 University of Hohenheim, Animal Genetics and Breeding (460g), Garbenstr. 17, Stuttgart 70599, Baden-Württemberg, Germany; 2 State Agency for Spatial Information and Rural Development Baden-Württemberg, Breeding Value Estimation Team, Stuttgarter Str. 161, Kornwestheim 70806, Baden-Württemberg, Germany

**Keywords:** migrant contribution, native contribution, native kinship, breeding costs, regional breed

## Abstract

Vorderwald cattle are a regional cattle breed from the Black Forest in south western Germany. In recent decades, commercial breeds have been introgressed to upgrade the breed in performance traits. On one hand, native genetic diversity of the breed should be conserved. On the other hand, moderate rates of genetic gain are needed to satisfy breeders to keep the breed. These goals are antagonistic, since the native proportion of the gene pool is negatively correlated to performance traits and the carriers of introgressed alleles are less related to the population. Thus, a standard Optimum Contribution Selection (**OCS**) approach would lead to reinforced selection on migrant contributions (**MC**). Our objective was the development of strategies for practical implementation of an OCS approach to manage the MC and native genetic diversity of regional breeds. Additionally, we examined the organisational efforts and the financial impacts on the breeding scheme of Vorderwald cattle. We chose the advanced Optimum Contribution Selection (**aOCS**) to manage the breed in stochastic simulations based on real pedigree data. In addition to standard OCS approaches, aOCS facilitates the management of the MC and the rate of inbreeding at native alleles. We examined two *aOCS* strategies. Both strategies maximised genetic gain, while strategy (I) conserved the MC in the breeding population and strategy (II) reduced the MC at a predefined annual rate. These two approaches were combined with one of three flows of replacement of sires (*FoR* strategies). Additionally, we compared breeding costs to clarify about the financial impact of implementing aOCS in a young sire breeding scheme. According to our results, conserving the MC in the population led to significantly (*P* < 0.01) higher genetic gain (1.16 ± 0.13 points/year) than reducing the MC (0.88 ± 0.10 points/year). In simulation scenarios that conserved the MC, the final value of MC was 57.6% ± 0.004, while being constraint to 58.2%. However, reducing the MC is only partially feasible based on pedigree data. Additionally, this study proves that the classical rate of inbreeding can be managed by constraining only the rate of inbreeding at native alleles within the aOCS approach. The financial comparison of the different breeding schemes proved the feasibility of implementing aOCS in Vorderwald cattle. Implementing the modelled breeding scheme would reduce costs by 1.1% compared with the actual scheme. Reduced costs were underpinned by additional genetic gain in superior simulation scenarios compared to expected genetic gain in reality (+4.85%).

## Implications

Vorderwald cattle are a regional cattle breed that has been crossbred with commercial breeds for upgrading in milk and meat performance. This led to decreasing originality and native genetic diversity of the breed. Advanced Optimum Contribution Selection facilitates the management of those parameters and simultaneously maximises the genetic gain. However, the implementation of advanced Optimum Contribution Selection is impeded by practical limitations. Thus, we developed solutions to practically implement advanced Optimum Contribution Selection in small-scale breeding schemes of regional breeds. Eventually, this could contribute to the preservation of the agrobiodiversity.

## Introduction

Vorderwald cattle are a livestock breed from the Black Forest in south western Germany. Despite the moderately low performance of the breed, it is well adapted to the local landscape, has a cultural value and contributes to the agricultural biodiversity. Over the past 50 years, the breeding program focused on performance traits and ignored the value of maintaining the genetic originality of the breed. This led to increased introgression from commercial breeds for upgrading (Hartwig *et al.*, 2014). Introgressed alleles replaced native gene variants and lowered the probability that alleles at a locus are identical by descent. Thus, genetic gain (*ΔG*) was achieved at a low rate of inbreeding (*Δf*) (Kohl *et al.*, 2019) but at the expense of decreasing genetic originality. The loss of genetic diversity can be measured in two different ways using: (I) the effective population size (*N*_*e*_), which is the number of individuals in an idealised population under random mating that would cause the same amount of decrease in genetic diversity as the population under study and (II) the native effective population size (*N*_*e(nat)*_), which is the corresponding parameter that quantifies how fast the genetic diversity at native alleles is decreasing (Wellmann *et al.*, 2012). Vorderwald cattle actually have an *N*_*e*_ of 102 but an *N*_*e(nat)*_ of only 34. This means that genetic diversity is high and the breed is vital in the long term, but only little of its genetic diversity is explained by native gene variants (Hartwig *et al.*, 2014). Huge amounts of irreplaceable genetic resources have passed genetic bottlenecks. Genetic bottlenecks for the genetic diversity at native alleles are caused by several factors, which include (I) direct displacement of native alleles by introgressed alleles and (II) directional selection for performance traits within the mixture of native and introgressed alleles. Today, performance traits are drastically improved in commercial breeds, and consumer demands on animal welfare are rapidly growing. This leads to changes in livestock breeding with traits like robustness, fitness and fertility being increasingly focused. Selection for *ΔG* led to effective population sizes as low as 52 in commercial breeds like German Holstein (Koenig and Simianer, 2006). Thus, alleles with large effects on new traits have already passed genetic bottlenecks in commercial breeds (Woolliams *et al.*, 2015). This will impede the selection for new traits. The additional genetic diversity that can be found in local breeds like Vorderwald cattle is mandatory to safeguard future changes in livestock breeding. As commercial breeds like Red Holstein and Montbéliard have already been introgressed to Vorderwald cattle (Hartwig *et al.*, 2014), there is a need for the management of introgressed genetic material. Optimum Contribution Selection (**OCS**) has been the standard to maximise *ΔG* and simultaneously manage *Δf* (Meuwissen, 1997). However, in breeds with historical introgression for upgrading reasons, OCS would lead to reinforced selection on introgressed alleles, since these are rare in the population and positively correlated to *ΔG* (Wellmann *et al.*, 2012). Displacement breeding would be the consequence. This is why Wellmann *et al.* (2012) invented an advanced Optimum Contribution Selection (**aOCS**) method, which facilitates the simultaneous management of *ΔG*, *Δf*, the rate of inbreeding at native alleles (*Δf*_*(nat)*_) (Wellmann *et al.*, 2012) and the amount of introgressed genetic material, that is, migrant contributions (**MC**). The MC is the portion of alleles descending from other breeds. The MC calculated from pedigree data is the expected percentage of introgressed alleles carried by an individual. On a population level, the MC already exceeds 60% in Vorderwald cattle. Therefore, starting the management of MCs is imperative. This study continues the investigation of Kohl *et al.* (2019). In that preceding article, a deeper insight in the underlying simulation process with explanations of the iterative phenotyping and breeding value estimation of the population under study is given. We examined opportunities to manage the MC with aOCS in Vorderwald cattle until marker data are available. In Germany, most regional breeds cannot afford the costs of genotyping. Additionally, the populations are frequently too small to build proper reference populations. Breeds with related gene pools could extent the reference populations with additional genotype data. However, genotyping and development of methodologies is outstanding. Hence, the genomic management of regional breeds is not widespread. Many regional breeds like Vorderwalder, Hinterwalder, German Angler and German Gelbvieh have to deal with historic migration for upgrading in performance traits (Bennewitz and Meuwissen, 2005). Thus, a pedigree-based aOCS approach is of great interest to manage the native genetic diversity and originality of those breeds till marker data are affordable and methodologies are developed. The objectives of this study were (I) the development of strategies for practical implementation of an aOCS approach to manage the MC and *Δf*_*(nat)*_ in breeding schemes of regional cattle breeds and (II) to examine the financial effects of implementing aOCS in connection with the modelled breeding scheme of Kohl *et al.* (2019) in the Vorderwald cattle breed. On one hand, conservation or reduction of the MC is needed. On the other hand, moderate rates of *ΔG* are mandatory to satisfy breeders to keep the breed. This is why we tested two different *aOCS* strategies: (I) keeping the MC at its current value while maximising *ΔG* at a given *Δf*_*(nat)*_ and (II) gradual displacement of the MC by native genetic material with an annual rate of −0.35% while maximising *ΔG* at a given *Δf*_*(nat)*_. Introduction of an aOCS approach to an existing breeding scheme requires modifications. Especially in regional breeds like Vorderwald cattle, where natural service is an important reproduction technique (50%). This is why we used *ZPLAN +* (Täubert *et al.*, 2010) to examine the financial effects of the modelled breeding scheme compared with the actual scheme of Vorderwald cattle.

## Material and methods

The aim of the stochastic simulation was to reflect reality and predict future developments of the breed by using aOCS under practical conditions. Since sufficient marker data are not yet available, and will not be in the foreseeable future, the simulations rely on pedigree data. A detailed description of the simulation protocol and the breeding scheme can be found in Kohl *et al.* (2019). The simulation process was iterated 21 times, whereby each iteration equalled 1 year. This reflects four generations with overlapping generations, on basis of a generation interval of 5.41 (Hartwig *et al.*, 2013). This timeframe was chosen, because we assume that marker data should be available afterwards. Further opportunities of aOCS by utilising marker data have already been examined by Wang *et al.* (2017).

### Base population

The raw data set, provided by the State Agency for Spatial Information and Rural Development Baden-Württemberg, consisted of 354 451 individuals with information on sex, breed, date of birth and estimated breeding values (**EBV**s) for the total merit index (**TMI**). Vorderwald cattle are a dual-purpose breed. The actual TMI is composed of performance traits in 44%, 44% and 12% for milk yield, fitness and meat production, respectively. Thus, we assumed a breed-specific TMI based on an individual’s own performance, equivalent to a simulated trait with *h*^2^ = 0.25 for our simulations (Kohl *et al.*, 2019). In Germany, EBVs are standardised with mean 100 and a genetic SD of 12. The iterative estimation of breeding values within the simulation process was modelled to reflect this variation and enable changes in EBVs due to increasing accuracies (*r*) over time. We processed the raw data set for individuals born before 2012 with at least three equivalent complete generations (MacCluer *et al.*, 1983). Pruning the raw data set for these individuals resulted in a pedigree of 89 911 individuals born between 1938 and 2012. Individuals born before 1970 and with unknown pedigree were defined as native founders and had an MC of 0%. Individuals from foreign breeds and individuals with unknown pedigree born after 1970 were defined as migrant founders with an MC of 100%. This assumption is based on the results of Hartwig *et al.* (2014) who found that severe introgression of foreign breeds to Vorderwald cattle started in 1970. After defining native and migrant founders, the MC was calculated for all individuals in the pedigree. Birth cohorts 2004 to 2012 consisted of 3 372 individuals on average. Therefore, the simulation process was designed to create 3 372 progeny per year, assuming that the population size remains constant.

### Selection candidates

Each year of the simulation started with sampling of deceased individuals (Kohl *et al.*, 2019). Selection candidates were labelled as purebred in the herd book of Vorderwald cattle. As aOCS was applied for overlapping generations, we defined concrete birth cohorts which spanned 1 year. Birth cohort *B*_*t*_ consisted of living individuals born in year *t*. Available proven sires for broad deployment were aged between 3 and 15 years, so these belonged to birth cohorts *B*_*t*−3_ to *B*_*t*−15_. Old sires were replaced by young sires, which completed 10 matings for progeny testing in *B*_*t*−1_. As a result, those had accuracies of EBVs of ≈0.5 (Kohl *et al.*, 2019) by the time of getting available for broad deployment in *B*_*t*−3_. The flow of replacement of sires (*FoR* strategy) has a significant impact on *ΔG* when aOCS is implemented (Kohl *et al.*, 2019). This is why we tested three different strategies*:* 10, 20 or 30 young sires for annual restock (*FoR10*, *FoR20*and *FoR30* strategies, respectively). Living dams belonging to birth cohorts *B*_*t*−1_ to *B*_*t*−9_ were available for breeding.

### Advanced Optimum Contribution Selection strategies

The result of OCS approaches for overlapping generations is a vector **c**_*t*_ with the desired genetic contributions **c**_*it*_ of each individual *i* to the next birth cohort *t* + 1. The genetic contribution of each individual *i* cannot be negative, that is, **c**_*it*_ ≥ 0. This is a general constraint for all OCS approaches. As a second constraint, the total genetic contributions of each sex equal 0.5, since the genes of diploid species originate 50% from sires and 50% from dams, that is, **c**_*t*_′**s**_*t*_ = 0.5 and **c**_*t*_′**d**_*t*_ = 0.5, where **s**_*t*_ and **d**_*t*_ are the indicative vectors of sex (0/1). Due to the limited number of progeny per cow and year, female contributions were forced to be equal, that is, **c**_*ti*_ = **c**_*tj*_ for all females *i*, *j*. Advanced OCS was only applied to the bull path. Since we face a breeding scheme with a substantial amount of natural service (≈50%), the maximum genetic contribution per male candidate was forced to not exceed 0.05, that is, **c**_*ti*_ ≤ 0.05 for all males *i*. As a result, aOCS selected at least 10 sires per year (**c**_*t*_*′***s**_*t*_*/*10 = 0.05) to satisfy the population under natural service. According to the absolute number of descendants per birth cohort, a single sire could service a maximum of 337 females per year (3372/10 ~ 337). Maximisation of *ΔG* can be achieved by maximising **c**_*t*_´**EBV**, where **EBV** is a vector of EBVs for TMI of the selection candidates. The maximisation of **c**_*t*_´**EBV**is done under constraints. Effective population sizes of 50 to 100 have been suggested in the literature to keep a breed vital in the long term (Meuwissen, 2009). Thus, we chose an *N*_*e(nat)*_ of 100 to be on the safe side. As an artefact of introgression, the classical kinship (*classKin*) is smaller than the native kinship (*natKin*). Restricting the *natKin* by an upper bound will automatically restrict the *classKin* and will manage both parameters simultaneously (Kohl *et al.*, 2019). We calculated the desired value for the annual increase in native kinship as

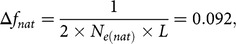

where *L* is the generation interval of 5.41. The first constraint of the aOCS procedure poses as an upper bound for *natKin* in the population at time *t* + 1:

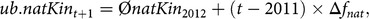

where 
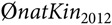
 is the average native kinship of the population in 2012. The second constraint poses as an upper bound for the MC in the population at time *t* + 1:

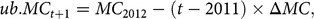

where MC_2012_ is the average MC of 58.2% of the base population in 2012 and *Δ*MC is the desired annual rate of decrease in MC. Our aim was to examine the possibilities of reducing the MC and the arising impact on *ΔG*. We tested two different *aOCS* strategies. Both strategies maximised *ΔG*. The MC was constrained in the first strategy as *Δ*MC = 0% to conserve the MC (*conserve-MC* strategy), and in the second strategy as *Δ*MC = −0.35% to reduce the MC annually (*reduce-MC* strategy). *Δ*MC of −0.35% was identified as the maximum value, for which most simulation scenarios solved the optimisation problem within the chosen time frame of four generations. Both *aOCS* strategies were combined with all *FoR* strategies, so six different scenarios were examined in total. Each scenario was replicated five times. The presented results are means averaged over replicates.

### Genetic gain

For a better interpretation of the results, we calculated *ΔG* as the average annual improvement in the mean EBV of the birth cohorts between year *t*_1_ and *t*_2_ as:

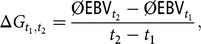

where 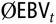 is the average EBV of birth cohort *t*, averaged over five replicates. To verify average value differences, we performed a two–factorial ANOVA and a Tukey honestly significant difference (**HSD**) test. The response variable was *ΔG*_2012,2033_. The *FoR* strategy and the *aOCS* strategy were used as factor variables. The development of *ΔG*in the real population was included for comparison. The average *ΔG* of real data between 2005 and 2015 was 1.18 points/year. Hence, we assumed that the *ΔG* will evolve linearly with 1.18 points/year in reality.

### Rates of inbreeding

We calculated *Δf* and *Δf*_*nat*_ for a time interval spanning from year *t*_1_ to *t*_2_ as

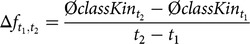

and

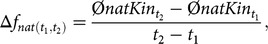

whereby the average was taken over five replicates.

### Migrant contributions

We calculated *Δ*MC as the average annual change of the mean MC of the birth cohorts for the time interval from year *t*_1_ to *t*_2_ as:

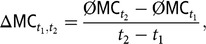

where 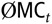 is the average MC of a birth cohort *t*, averaged over five replicates.

### Number of deployed sires

Since natural service is an important reproduction technique in Vorderwald cattle, the number of annually contributing sires influences the feasibility of a future breeding scheme. This is why we calculated the average number of annually contributing sires:

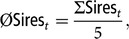

where 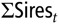 is the sum of sires that contributed to birth cohort *t*, averaged over five replicates.

### Breeding costs

We used the software *ZPLAN+* (Täubert *et al.*, 2010) to calculate breeding costs of the young sire breeding scheme (Kohl *et al.*, 2019) in connection with a *FoR23* strategy and 10 matings for progeny tests per young bull. The calculated costs of the young sire breeding scheme were compared with the costs of the actual scheme of Vorderwald cattle (Kohl *et al.*, 2019). Reasons for choosing a *FoR23* strategy rather than a *FoR10*, *FoR20* or *FoR30* strategy will be given in the discussion (see the “Transfer to a practical breeding scheme” section). *ZPLAN+* utilises a deterministic evaluation of breeding schemes. It is based on selection index theory (Hazel and Lush, 1942) and geneflow (Hill, 1974) in populations with overlapping generations. The results of *ZPLAN+* provide population parameters, discounted costs and discounted returns of a breeding scheme. However, the results of *ZPLAN+* were exclusively used to estimate breeding costs per animal and year, because population parameters and discounted returns are based on selection index theory rather than aOCS-based simulation results. Alternative software to evaluate various breeding schemes are *SelAction* (Rutten *et al.*, 2002) and *ADAM* (Pedersen *et al.*, 2009). The output of *SelAction* includes the response to selection measured in economic units (Rutten *et al.*, 2002). The software *ADAM* uses stochastic simulations to evaluate selective breeding schemes. However, *SelAction* and *ADAM* do not facilitate a monetary evaluation of the costs of different schemes. Hence, we chose *ZPLAN+* for our study. The costs of a breeding scheme are composed of fix and variable costs. The fix costs of the actual breeding scheme and the young sire scheme were assumed to be equal. The variable costs included: milk recording (33€/year/cow), selection of bull calves (10€/calve), performance tests of bull calves (50€/bull), keeping of waiting bulls (100€/ bull/year), evaluation of daughters (50€/daughter), production of straws (1€/straw) and storage of straws (0.03€/straw) (Priv. Doz. Dr. Pera Herold, personal communication, 10 May 2018). The sum of all breeding costs is covered by each individual of a breeding scheme. Thus, the total breeding costs were divided by the number of individuals (Vereinigte Informationssysteme Tierhaltung w.V., 2011).

## Results

### Genetic gain

**Figure 1:**
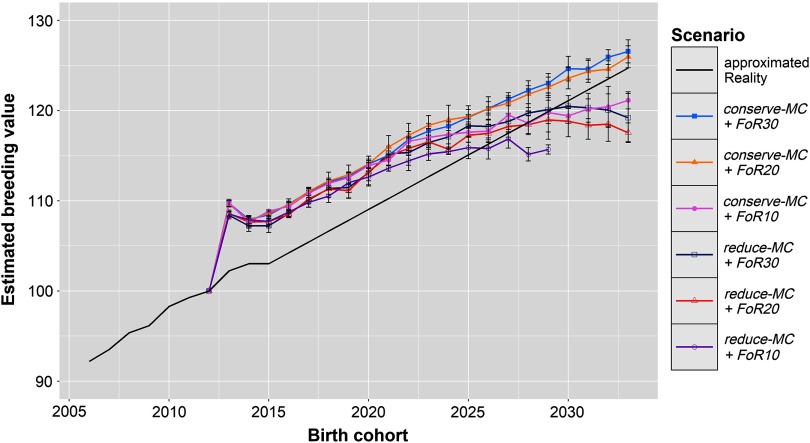
Development of genetic gain of birth cohorts in different simulation scenarios and extrapolated reality for Vorderwald cattle – Development of mean estimated breeding values for the total merit index of birth cohorts in simulation scenarios and reality. Development in the real population was calculated based on the real data between 2005 and 2015. We assumed that the genetic gain will evolve linearly with an annual rate of 1.18 points/year. In simulation scenarios, we examined varying flows of replacement of sires with 10, 20 or 30 on an annual basis (*FoR10*, *FoR20* and *FoR30*, respectively) in combination with two different *aOCS* strategies. The first one conserved the mean MC in subsequent birth cohorts (*conserve-MC*). The second one reduced MC with an annual rate of –0.35% (*reduce-MC*). Results of simulation scenarios were averaged over five replicates (±*SD*). aOCS = advanced Optimum Contribution Selection; MC = migrant contribution.

Figure 1 shows the development of *ΔG* in the simulation scenarios and the calculated *ΔG* in reality. All simulations show a strong increase of the EBVs in the first birth cohort (*ΔG*_2012,2013_ = + 9.1 ± 0.7), which was followed by slightly lower EBVs in the following two birth cohorts (*ΔG*_2013,2015_ = −0.51 ± 0.32). After 2015, the EBVs increased linearly in scenarios *conserve-MC + FoR10*, *conserve-MC + FoR20* and *conserve-MC + FoR30* with *ΔG*_2012,2033_ of 1.01 ± 0.05, 1.24 ± 0.06 and 1.27 ± 0.06, respectively. In scenarios *reduce-MC + FoR20* and *reduce-MC + FoR30*, *ΔG*_2015,2029_ (0.80 ± 0.14 and 0.92 ± 0.10, respectively) was significantly higher (*P* = 9 × 10^−10^) than the annual increase in the following birth cohorts (*ΔG*_2029,2033_ = −0.36 ± 0.36 and −0.22 ± 0.32, respectively). As a result, *ΔG*_2012,2033_ was 0.84 ± 0.05 in *reduce-MC + FoR20* and 0.92 ± 0.13 in *reduce-MC + FoR30*. The optimisation problem could not be solved in 2029 for scenario *reduce-MC + FoR10* because no solution existed. However, *ΔG*_2012,2029_ was 0.91 ± 0.05. According to the Tukey HSD test, *ΔG*_2012,2033_ was significantly affected by the *aOCS* strategy and by the *FoR* strategy (*P* = 2 × 10^−11^ and 2 × 10^−6^, respectively). Conserving the MC realised higher *ΔG*_2012,2033_ (1.16 ± 0.13) than reducing the MC (0.88 ± 0.1). The *FoR10* strategy produced lower *ΔG*_2012,2033_ (1.0 ± 0.05) than the *FoR20* and *FoR30* strategy (1.04 ± 0.22 and 1.09 ± 0.21, respectively). The lower variation of *ΔG*_2012,2033_ in *FoR10*scenarios is reasoned in the missing results of scenario *reduce-MC + FoR10* in 2033. However, we performed an additional Tukey HSD test for *ΔG*_2012,2029_ that proved present results. Additionally, *conserve-MC + FoR20* and *conserve-MC + FoR30* produced a higher *ΔG*_2012,2033_ than calculated for reality. Other scenarios created less (Figure 1 and Table 1).

**Table 1:** Different simulation scenarios are explained by a combination of implemented aOCS strategy and FoR strategy of Vorderwald cattle

Scenarios	*FoR*^1^	*aOCS*^2^	*n*	*ΔG*_2012 to 2033_^3^	*Δf*_*nat*_	*N*_*e(nat)*_	*Δf*	*N*_*e*_	MC_2033_
Reality	≈2 to 3^4^	TS	1	1.18^5^	NA^6^	NA	NA	NA	NA
*Conserve-MC* + *FoR10*	10	*Conserve-MC*	5	1.01 ± 0.05^AX^	0.092 ± 0.0004	100.8	0.097 ± 0.002	94.6	57.8 ± 0.18
*Conserve-MC* + *FoR20*	20	*Conserve-MC*	5	1.24 ± 0.06^BX^	0.091 ± 0.0006	101.3	0.099 ± 0.004	93.2	57.8 ± 0.28
*Conserve-MC* + *FoR30*	30	*Conserve-MC*	5	1.27 ± 0.06^BX^	0.091 ± 0.0005	101.5	0.093 ± 0.004	99.3	57.3 ± 0.51
*Reduce-MC* + *FoR10*	10	*Reduce-MC*	5	NA/0.91 ± 0.05^7^^AY^	0.092 ± 0.0007	101.0	0.090 ± 0.002	103.1	NA/ 50.7 ± 0.29^7^
*Reduce-MC* + *FoR20*	20	*Reduce-MC*	5	0.84 ± 0.05^BY^	0.091 ± 0.0007	101.8	0.083 ± 0.002	111.8	49.4 ± 0.10
*Reduce-MC* + *FoR30*	30	*Reduce-MC*	5	0.92 ± 0.13^BY^	0.091 ± 0.0004	101.9	0.081 ± 0.001	114.0	49.2 ± 0.23

Scenarios = different scenarios are explained by a combination of *FoR* strategy and *aOCS* strategy; aOCS = advanced Optimum Contribution Selection; *FoR* = annual flow of replacement of sires; *n* = replicates per scenario; *ΔG* = genetic gain; *Δf_nat_* = rate of native Inbreeding for overlapping generations per year. Restricted to 0.092; *N_e(nat)_* = native effective population size; *Δf* = rate of Inbreeding for overlapping generations per year. Not restricted by aOCS; *N_e_* = effective population size; TS = truncation selection; MC = migrant contribution; MC_2034_ = Average migrant contribution of birth cohort 2033 as final value.

1 Three different *FoR* strategies were examined with 10, 20 or 30 young sires for restock per year.

2 Two different *aOCS* strategies were examined. Either conserving or reducing MCs in the next birth cohort with an annual rate of 0.0% or –0.35%, respectively.

3 Genetic gain was defined as improvement in mean estimated breeding values for the total merit index among birth cohorts *B*_2012_ to *B*_2033_.

4 According to personal communication (Dr Franz Maus, 22 February 22 2018).

5 Genetic gain in reality was calculated based on the real data between 2005 and 2015.

6 NA = Not available.

7 *Reduce-MC+ FoR10* was the only simulation scenario for which the aOCS optimisation problem could not be solved in 2029. Thus, NAs relate to 2033. The given figure relates to 2029.

^A,B,X,Y^Different superscripts label significantly different values at *P* < 0.01 in terms of *FoR* strategies (A *v.* B) or *aOCS* strategies (X *v.* Y).

### Migrant contributions

**Figure 2:**
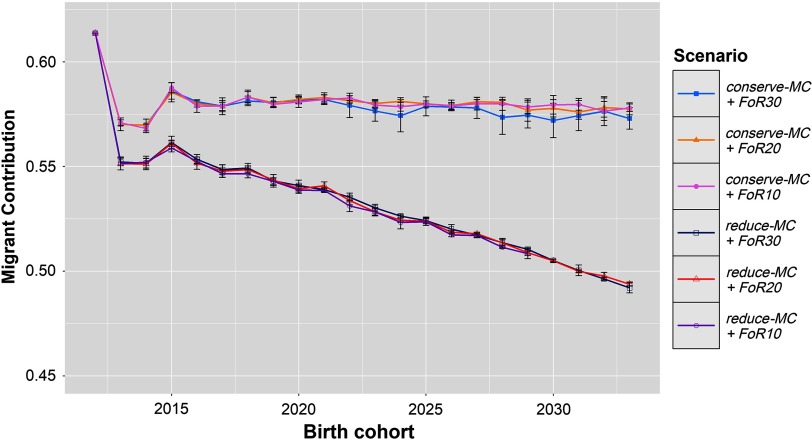
Development of MCs of birth cohorts in different simulation scenarios for Vorderwald cattle – Development of mean MC of birth cohorts in simulation scenarios. We examined varying flows of replacement of sires with 10, 20 or 30 on an annual basis (*FoR10*, *FoR20* and *FoR30*, respectively) in combination with two different *aOCS* strategies. The first one conserved the mean MC in subsequent birth cohorts (*conserve-MC*). The second one reduced MC with an annual rate of –0.35% (*reduce-MC*). Results of simulation scenarios were averaged over five replicates (±*SD*). MC = migrant contribution; aOCS = advanced Optimum Contribution Selection.

The development of the average MC of the birth cohorts is shown in Figure 2. In the *conserve-MC* scenarios, *Δ*MC_2012,2013_ was−4.33% ± 0.25. In the *reduce-MC* scenarios*, Δ*MC_2012,2013_ was −6.20% ± 0.25. This decline in MC was followed by an increase till 2015 in all scenarios (*Δ*MC_2013 to 2015_ = + 0.62% ± 0.27). After 2015, the MC evolved linearly and eventually reached 57.6% ± 0.004 and 49.3% ± 0.002 for *conserve-MC* and *reduce-MC* scenarios, respectively. As a result, the corresponding *Δ*MC_2012,2033_ were −0.18% ± 0.02 and −0.58% ± 0.01. Overall, the *reduce-MC* scenarios reduced the MC by 8.9% ± 0.002.

### Classical kinship and kinship at native alleles

**Figure 3:**
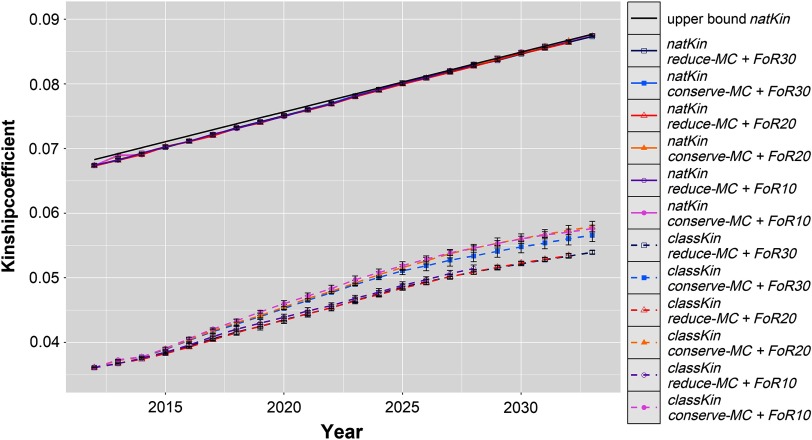
Development of average classical and native kinship coefficients of evolving populations in different simulation scenarios for Vorderwald cattle – We examined varying flows of replacement of sires with 10, 20 or 30 on an annual basis (*FoR10*, *FoR20* and *FoR30*, respectively) in combination with two different *aOCS* strategies. The first one conserved the mean MC in subsequent birth cohorts (*conserve-MC*). The second one reduced the MC with an annual rate of –0.35% (*reduce-MC*). Both *aOCS* strategies restricted the average kinship at native alleles (*natKin*) of the population by an upper bound (black and solid), meanwhile the classical kinship (*classKin*) was not managed. The graphs are subdivided for different simulation scenarios (colour) and both kinship coefficients (solid and dashed). Results of simulation scenarios were averaged over five replicates (±*SD*). aOCS = advanced Optimum Contribution Selection; MC = migrant contribution.

The realised *Δf*, *Δf*_*nat*_ and the upper bound on *Δf*_*nat*_ are visualised in Figure 3. Across all simulated populations, the *Δf*_*nat*_ was 0.091 ± 0.0006 per year, which was only slightly below the constraint setting of *Δf*_*nat*_ (0.092 per year). *Δf* followed similar trends with 0.091 ± 0.007 across all scenarios, although it was not constrained by the aOCS procedures. As a result, the realised *N*_*e(nat)*_ and *N*_*e*_ were 101.4 ± 0.4 and 102.1 ± 8.7 across all simulated populations, respectively.

### Selected sires

**Figure 4:**
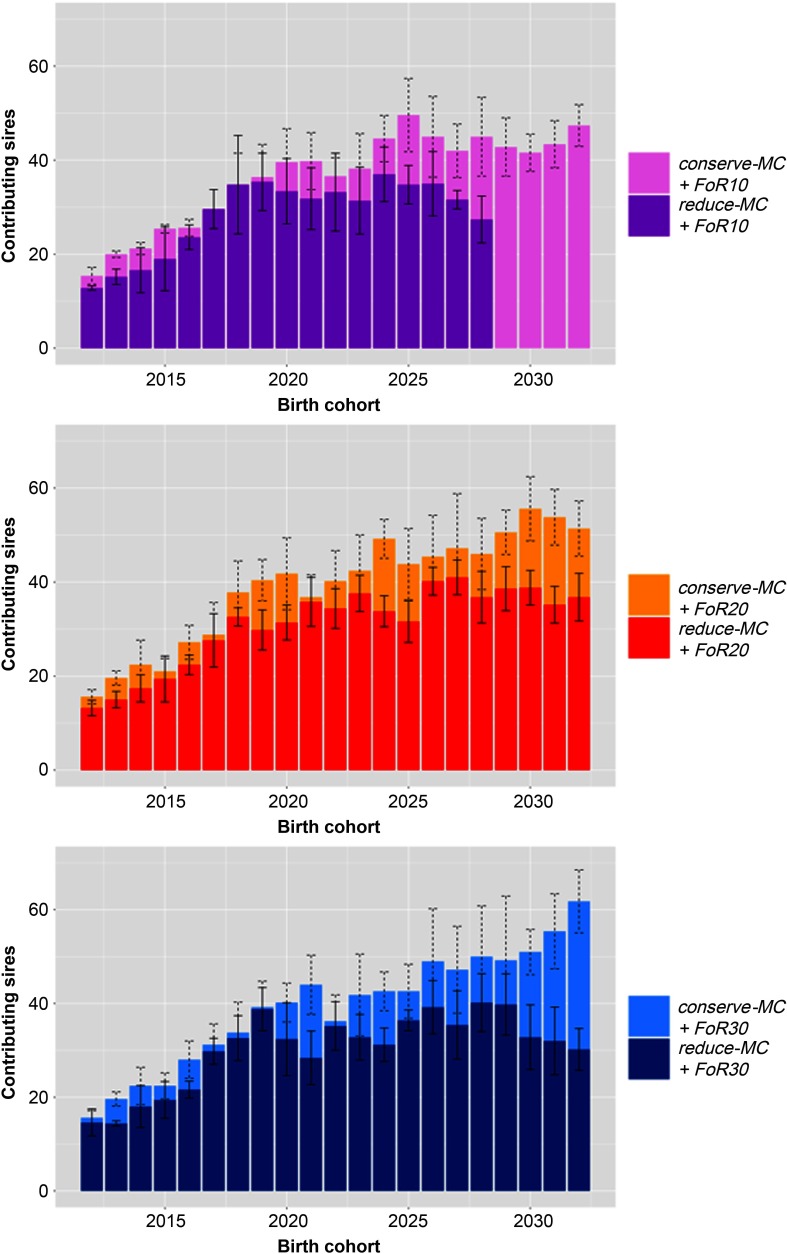
Bar graph of annually contributing sires in different simulation scenarios for Vorderwald cattle – We examined varying flows of replacement of sires with 10, 20 or 30 on an annual basis (*FoR10*, *FoR20* and *FoR30*, respectively) in combination with two different *aOCS* strategies. The first one conserved the mean MC in subsequent birth cohorts (*conserve-MC*). The second one reduced MC with an annual rate of –0.35% (*reduce-MC*). The bar graphs are subdivided for *FoR* strategies. Bars visualise the average of annually contributing sires averaged over five replicates (±*SD*). aOCS = advanced Optimum Contribution Selection; MC = migrant contribution.

The mean number of sires that contributed to the birth cohorts is visualised in Figure 4. Different bar graphs are shown for the different *FoR* strategies. In all scenarios increasing numbers of sires contributed to the birth cohorts at the beginning of the simulation with *øSires*_2012_ = 14.6 ± 2.0, *øSires*_2015_ = 21.4 ± 4.3 and *øSires*_2020_ = 36.5 ± 7.2. Subsequently, the number of contributing sires was rather constant with *øSires*_2020 to 2033_ = 46.2 ± 3.6 across *conserve-MC* scenarios and 29.7 ± 6.0 across *reduce-MC* scenarios.

### Breeding costs

We give a brief overview of costs of the young sire scheme in combination with the *FoR23* strategy. Costs are compared to the actual scheme to give a better understanding of the financial opportunities. According to the results of *ZPLAN+*, costs sum up to 18.44€/breeding animal/year and 18.23€/breeding animal/year for the actual breeding scheme and the young sire scheme, respectively. Thus, costs could be decreased by 1.1% by implementing aOCS in connection with the young sire scheme.

## Discussion

### Comparison of scenarios

The first two years belong to *Stage 1* of the simulation process of Kohl *et al.* (2019) in which no new male selection candidates became available. The EBVs increased strongly in all scenarios in the first birth cohort (*ΔG*_2012,2013_ = + 9.1 ± 0.7; Figure 1), while the MC decreased (*Δ*MC_2012,2013_ = −4.33% ± 0.25 and −6.20% ± 0.25; Figure 2). This reflects the huge short-term optimisation opportunities of aOCS in breeding schemes that formerly used truncation selection (Kohl *et al.*, 2019). Probably, the MC decreased because the most influential sires had by chance an MC that was slightly below average. In the following years, the EBVs of the birth cohorts were slightly smaller (*ΔG*_2013,2015_ = −0.51 ± 0.32) because keeping the desired level of the native kinship in the population required the use of less superior sires. These sires were chosen from the same pool as in 2012 because until 2015 no new male candidates were introduced. The number of contributing sires indeed increased from 2012 to 2015 (*øSires*_2012_ = 14.6 ± 2.0, whereas *øSires*_2015_ = 21.4 ± 4.3; Figure 4). The MCs increased slightly between 2013 and 2015 but were kept below the constraint setting (*Δ*MC_2013,2015_ = + 0.62% ± 0.27 in all scenarios). In 2015, first young sires got available for broad deployment, so the introduction of new proven sires finished the impediment of selection. Those were less related to the population and had on average higher EBVs compared to their older companions (results not shown). As expected, the EBVs increased linearly and the MCs were conserved in scenario *conserve-MC* from birth cohort 2015 onwards. The numbers of contributing sires further increased until ~2020 across all scenarios (*øSires*_2015_ = 21.4 ± 4.3 towards *øSires*_2020_ = 36.5 ± 7.2). Over the course of the simulation process, *Δ*MC_2012,2033_ were −0.58 ± 0.01 and −0.18% ± 0.02 for *reduce-MC* and *conserve-MC*, respectively, while the constraint setting for *Δ*MC were −0.35% and 0.0%, respectively. Thus, all scenarios reduced the MC at a higher rate than desired. This was an artefact of the random decrease of the MC in 2013.

All scenarios that selected for reduced MC reached a selection plateau for the EBVs around 2023, which was after the second generation was born. The reason is that pedigree data were used for estimating the MC. When pedigree data are used, then the MC of an individual is always the average of the MCs of its parents. Continued averaging of these values in newborn individuals narrows the range of the MCs in the population, and the mean of the MCs converges to a value which is above the minimum MC that was present in a selection candidate in 2012. Hence, the number of bulls whose MC surpasses the threshold value for MC is strongly decreasing after a few years. This reduces the number of males that can be used for breeding, which in turn reduces strongly the achievable selection intensity on the EBV for TMI. After some years of management, the OCS problem can no longer be solved. The optimisation problem could not be solved for scenario *reduce-MC + FoR10* in 2029 because not enough sires with low MC existed that had a sufficiently low native kinship with the population. The situation was slightly better in scenarios *FoR20* and *FoR30* because sires could be selected from a larger pool. The number of sires to select from is 130, 260 and 390 in *FoR10*, *FoR20* and *FoR30* scenarios, respectively. This shows clearly that pedigree data cannot be used longer than two generations to reduce the MC of a population. We assumed a breed-specific heritability for the TMI of *h*^2^ = 0.25 (Kohl *et al.*, 2019) to run the stochastic simulations. However, Gandini *et al.* (2014) reported that the *ΔG* will increase as heritabilities of traits increase when an OCS approach is implemented. Thus, the *ΔG* that will be realised by implementing aOCS in reality might deviate from the observed results. However, the utilised *h*^2^ = 0.25 is realistic with respect to the composition of the actual TMI of Vorderwald cattle.

### Deciding for superior strategies

According to the results of the Tukey HSD tests, *ΔG* was affected significantly by *aOCS* strategies (*P* = 2 × 10^−11^) as well as *FoR* strategies (*P* = 2 × 10^−6^). The *conserve-MC* scenarios resulted in a higher *ΔG* than the *reduce-MC* scenarios. The total selection intensity was the same in both scenarios, because the population sizes and the increases in the native kinship were equal (Figure 3). However, in the *conserve-MC* scenarios, selection was only on EBVs for TMI, whereas in the *reduce-MC* scenarios, selection was on both the EBVs and the MCs. The constraint for the MC became difficult to satisfy after a few years of selection because pedigree data were used to estimate the MC. Consequently, more and more of the selection intensity was allocated to reduce the MC and less of it was allocated to achieve genetic gain. Consequently, genetic gain was lower in the *reduce-MC*scenarios than in *conserve-MC* scenarios and eventually approached zero. Subsidies for breeders for conserving the breed instead of keeping commercial breeds are not sufficient to cover the expenses (Kohl and Herold, 2017). Thus, further economical disadvantages through a lower *ΔG* must be avoided. In a breeding program that reduces the MC, pedigree data must be replaced by genomic data after no more than two generations of selection because otherwise, the EBVs would soon reach a selection plateau. This reduced genetic gain could lead to a growing probability of extinction for the Vorderwald breed (Meuwissen, 2009). Conclusively, we recommend to implement the *conserve-MC* strategy until genotype data are available to reduce the MC. Genomic data enhance the aOCS approach by estimation of MC based on shared haplotype segments (runs of homozygosity) (Wang *et al.*, 2017). As soon as genotypes are accessible, further studies are needed to examine possibilities of reducing the MC in connection with an adjusted breeding scheme.

The *FoR20* and *FoR30* strategy resulted in a significantly higher *ΔG* than the *FoR10* strategy (*P* = 2 × 10^−6^). The highest *ΔG* was achieved by the *conserve-MC + FoR30* strategy but was not significantly different from the genetic gain of the *conserve-MC + FoR20* strategy. Since selection as well as husbandry and progeny testing of young sires is cost-intensive, we recommend to implement the *FoR20* strategy and conserve the MC.

### Transfer to a practical breeding scheme

The actual breeding scheme of Vorderwald cattle selected 42 bull calves annually to pass a performance test on station. Subsequently, 2 to 3 of them were selected to replace sires for artificial insemination after passing a progeny test (Kohl *et al.*, 2019). According to our results, the *FoR20* strategy is sufficient to maximise *ΔG* when aOCS is used. However, the simulation is based on the assumption that semen will be taken from all young sires. This is unrealistic because some of them will not be available at the time of production. Based on the results of Wathes *et al.* (2008), we assume that 10% of bull calves might pass away between selection (within 1st month) and progeny testing (≈12th month). Consequently, we recommend to select 22 to 23 bull calves for a performance test on station. Subsequently, all surviving young bulls should pass a progeny test and produce straws, irrespective of their performance. Thus, at least 20 young sires will be available for annual restock of proven sires. Conclusively, the superior *FoR20* strategy of simulations is transferred to a practical breeding scheme by implementing a *FoR23* strategy in reality. This is why we compared costs of the actual breeding scheme to a young sire scheme in connection with a *FoR23* strategy in *ZPLAN+*.

Furthermore, we highly recommend to accelerate the implementation of selection of elite dams. This would facilitate directed mating with sires of sires. Selection of dams should be optimised by a modified aOCS approach that additionally optimises female contributions. Consequently, bull calves could be selected not only in the broad population but also among descendants of elite matings (Kohl *et al.*, 2019). This would result in three benefits: (I) Further enhanced population parameters (Gandini *et al.*, 2014). (II) Unavailability of bull calves at the time of selection (sale and culling) could be avoided. (III) Simplified selection of bull calves, since dams are known. Currently, 3 160 dams are serviced naturally (Kohl *et al.*, 2019). This is a further obstacle to overcome. In the actual breeding scheme, 80 sires were available for that task. According to our results, aOCS deployed 39.9 ± 1.3 sires in *conserve-MC + FoR20*. This shows that fewer sires will be available for natural mating. To meet this practical constraint, we recommend to keep young sires alive after production of straws till birth cohort *B*_*t*−4_. Thus, those would be available for broad deployment of ≈2 years, i.e. parts of *B*_*t*−2_ (i.e., after progeny testing), *B*_*t*−3_ and *B*_*t*−4_. Hence, 40 to 60 young sires (replacement rate and mortality rate) will be available for natural service. The biggest challenge of implementing optimum contributions in the naturally serviced dam population is the organisation of stock bull availability in single herds. Kohl *et al.* (2019) proposed a stock-bull-to-herd rotation program. On one hand, the accuracy of breeding value estimation could be improved through enhanced estimation of herd effects. On the other hand, this will cause organisational challenges, since sharing stock bulls will compromise the hygienic status of herds. Several approaches are conceivable to overcome that problem. The first option might be to put stock bulls under quarantine as those arrive at each herd. However, this approach has some drawbacks: Reasonable quarantine periods of mammals should last for a minimum of 30 days (Miller, 1996). At best, one stock bull could serve 11 herds per year, by complying this quarantine period. However, as aOCS is introduced to a naturally serviced dam population, there is a need for several stock bulls per herd. Thus, practicability of this approach depends on the number of herds using natural service. As a second approach, we thought of a farm cooperative solution. Thus, consistent groups of farms would share stock bulls, minimising hygienic risks of single herds since their hygienic status should converge over time. However, quarantine periods would still be necessary as stock bulls are supplied from one farm cooperative to the other. Additionally, the necessity of introducing optimum contributions in farm cooperatives by usage of several stock bulls is still a problem. Small-scale breeders might have space problems related to the need of bull paddocks to keep unused bulls till these are delivered. Furthermore, it is unclear which farms should set up the quarantine facilities and who will bear the costs. As a result, we propose two approaches, where one of these should be chosen depending on the possibilities of the breeding organisation: (I) A bull leasing system with the breeding organisation as lessor. Since genomic breeding value estimation has conquered the livestock breeding environment, most breeding organisations still have huge properties where waiting bulls have formerly been kept. Those could be used to keep stock bulls to be leased by the breeders. Thus, the hygienic status of stock bulls would be equal and could be managed professionally. (II) Epidemic units, where higher scale breeders should set up quarantine facilities to keep several stock bulls and organise stock bull delivery to connected smaller scale breeders. In both approaches, hygienic concepts have to be developed by the breeding organisation in connection with the breeders themselves. Additionally, the increasing aggressiveness of eldering stock bulls is a well-known problem among practitioners. This is the main reason of restricting the usage of stock bulls to 4 years of age. This restriction is flexible as it is subjective and might be breed-specific. Additionally, the increasing aggressiveness of eldering stock bulls could be a new trait to be implemented in the breeding objective of Vorderwald cattle. Further studies are needed to examine the possibilities for that. Schreiner (2018) has shown that higher costs of required measures on farm distract breeders to sign conservation breeding contracts. Additionally, the payout structures of subsidies for conserving genetic agrobiodiversity at Germany are too inflexible. Breeding organisations are legal entities. Thus, those are disqualified from subsidies by the ‘GAK-Förderzahlungen’ (Bundesministerium für Ernährung und Landwirtschaft, 2016). The subsidies for breeders are exclusively connected to keeping a breeding animal and participation in a corresponding breeding scheme. This problem has been outspoken several times, but political actions are still missing. Required measures, that is, setting up quarantine facilities or a bull leasing system, to effectively conserve endangered livestock breeds should be subsidised separately. In reality, breeders are discouraged to invest in required measures without having a financial benefit. Thus, applicability of the modelled breeding scheme is questionable till the funding of required measures is clarified. Additionally, Schreiner (2018) mentioned that monitored pairing discourages breeders to sign conservation breeding contracts. However, the underlying aOCS approach optimises exclusively the contributions of sires to the following birth cohort. The selection in the dam path and the pairing remains a core competence of breeders. However, the pairing decisions of breeders will be compromised by the estimated optimum contributions of sires. As soon as the estimated contribution of a sire reaches the optimum, the breeding organisation should stop supplying the corresponding semen and stock bulls within the chosen time frame. In addition to the scientific work of this study, we had participative meetings about the aOCS methodology and necessary adjustments to the breeding scheme to sensitise the breeders. Marsoner *et al.* (2018) investigated the socio-ecological and cultural value of indigenous breeds. The authors reported about the maintenance of cultural landscapes, the contribution to cultural heritage and identity and the promotion of tourism through locally adopted breeds. The Vorderwald cattle breed contributes to those benefits in the region of the Schwarzwald in Germany. Thus, the lost profits of keeping Vorderwald cattle, instead of a commercial breed, were tolerated among breeders so far. Additionally, the breeders of Vorderwald cattle obtain subsidies for conserving the native genetic background of an endangered breed, although the MC of Vorderwald cattle already exceeds 60% with an *N*_*e(nat)*_ of 34. Eventually, the breeders might accept the compromises on breeding decisions. The lost profits have already been accepted in the past, and the effective management of the MC is imperative to not lose the eligibility for future subsidies.

### Breeding costs

*ZPLAN+* was used to compare costs of the actual breeding scheme and the young sire scheme in connection with a *FoR23* strategy and 10 matings for progeny testing of each young sire. Since *ZPLAN+* is based on selection index theory, population parameters were omitted. The actual breeding scheme of Vorderwald cattle accepts accuracies of EBVs of young sires depending on 25 to 30 daughters (Hartwig *et al.*, 2013). Actually, 250 to 300 matings are required for progeny tests of 2 to 3 young sires annually. According to Kohl *et al.* (2019), 14 to 15 matings per young sire are sufficient to maximise *ΔG* in the broad population when aOCS is used. Therefore, progeny tests of 23 young sires would result in 322 to 345 matings in total. Thus, the number of matings for progeny testing, and subsequent costs, increases by 15% to 30%. However, the *FoR23* strategy will reduce performance tests of bull calves on station (23 towards 42). The arising savings of reduced performance tests will overcompensate the expenses of increased progeny testing. As a result, implementing aOCS with the recommended superior strategies will reduce costs by 1.1%, according to *ZPLAN+*. Finally, a comparison of expected *ΔG* underpins financial benefits. The rate of *ΔG* of *conserve-MC + FoR20* was 4.85% higher compared to extrapolated *ΔG* in reality. Unfortunately, accurate values for monetary assessment of *ΔG* are not available for Vorderwald cattle. However, financial benefits will further expand.

### Behind practical aspects

Behind practical aspects, data flow, work flow and accountable actors of the breeding scheme have to be defined. In most cases, the breeding organisation is responsible for registration of breeding animals in the stud book. At Baden-Württemberg in Germany, breeding value estimation is a state-owned task. Therefore, the breeding organisation of Vorderwald cattle supplies studbook data to governmental authorities. Following the breeding value estimation, aOCS should be conducted. Two options are conceivable here. On one hand, aOCS could be implemented at the governmental authority that drives the breeding value estimation. In this way, the breeding organisation has to supply additional data that identify the living dam population and available sires. The backflow of data would contain EBVs, optimum contributions of breeding animals and selection proposals for elite animals calculated by aOCS (dams of sires and sires of sires). As an opportunity of this option, all calculation and estimation tasks are consolidated. A drawback is the more complicated data flow and the need for communication paths, as results of the aOCS approach might seem questionable sometimes. On the other hand, governmental authorities could supply merely the EBVs to the breeding organisation (as done so far). In this way, aOCS would be conducted at the breeding organisation itself. As an opportunity of this approach, data division would remain the same and questions about results of the aOCS approach could be scrutinised within the breeding organisation itself. Normally, all of the mentioned tasks are consolidated within the breeding organisation. Thus, the data flow, work flow and appointment of accountable actors are an internal issue of the breeding organisation.

### Additional information

In this study, we used

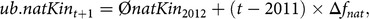

as the upper bound for *natKin* in birth cohort *t* + 1. An alternative would be to use

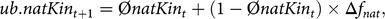

where 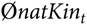 is the mean native kinship of selection candidates in year *t*. However, this led to a bias because matings for progeny tests of young sires were not optimised by aOCS, although these contribute to 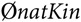 in year *t* + 1. Thus, 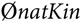 increased at another rate than expected. This bias in *ub.natKin*_*t* + 1_ would accumulate over the years. As a result, *N*_*e(nat)*_ would vary between 78 and 93, although it should have been constrained to 100. To be on the safe side and avoid an accumulation of errors over the years, *ub.natKin*_*t*_ should be constrained as done in this study.

Additionally, this study proves the possibility of controlling *Δf* by setting an upper bound only for *Δf*_*nat*_. Values for *N*_*e*_ varied within 93.2 to 114.0 across all scenarios. Despite the large variation of *N*_*e*_ across scenarios, the variation within scenarios was rather small (Table 1, SDs of *Δf*). Effective population sizes of 50 to 100 have been suggested to keep a breed vital in the long term (Meuwissen, 2009). Thus, restricting only *Δf*_*nat*_ is sufficient to manage the genetic diversity of the breed.

## Conclusion

The examined scenarios and the corresponding breeding costs revealed the potential to reduce the MC with pedigree data and the potential of implementing aOCS in Vorderwald cattle in connection with a young sire breeding scheme. Replacing pedigree-based estimates of the MC by genomic estimates after no more than two generations of selection on reduced MC turned out to be mandatory for a breeding program that aims at recovering the native genetic background of a breed. From a conservational perspective, it would be desirable to implement the *reduce-MC* strategy. However, subsidies for keeping the breed are not sufficient to compensate expenses. Thus, the *ΔG* should not decrease in comparison to the actual population parameters. Implementation of the *conserve-MC + FoR23* strategy would create sufficient *ΔG*, manage *Δf*_*nat*_ at the desired level, *conserve-MC* and keep costs within an acceptable range. Unfortunately, the implementation of aOCS to an existing breeding scheme presumes investments in required measures. The funding of those measures has to be clarified before. Additionally, the freedom of breeding decisions by breeders will be restricted by the estimated optimum contributions of sires. Eventually, the funding of required measures and the required contracts with breeders might be the biggest obstacles to overcome when implementing aOCS in a practical breeding scheme.

## Software and data repository resources

The data and the simulation code will be archived at the State Agency for Spatial Information and Rural Development Baden-Württemberg at Kornwestheim, Germany. Access might be given after a consultation with the Rinderunion Baden-Württemberg e.V. and the Breeding Value Estimation Team Baden-Württemberg.
